# *WNT5A* Encodes Two Isoforms with Distinct Functions in Cancers

**DOI:** 10.1371/journal.pone.0080526

**Published:** 2013-11-18

**Authors:** Matthieu Bauer, Jean Bénard, Terry Gaasterland, Karl Willert, David Cappellen

**Affiliations:** 1 Centre National de Recherche Scientifique-Université Paris, Institut de Cancérologie Gustave Roussy, Villejuif, France; 2 Department of Cellular and Molecular Medicine, University of California San Diego, La Jolla, California, United States of America; 3 Département de Pathologie et Biologie Médicales, Institut de Cancérologie Gustave Roussy, Villejuif, France; 4 University of California San Diego and Scripps Institution of Oceanography, La Jolla, California, United States of America; 5 Centre Hospitalier Universitaire de Bordeaux, Université Bordeaux, Bordeaux, France; 6 Histologie et Pathologie Moléculaire des Tumeurs, Université Bordeaux, Bordeaux, France; Northwestern University Feinberg School of Medicine, United States of America

## Abstract

WNT5A, a member of the WNT family of secreted lipid-modified glycoproteins, is a critical regulator of a host of developmental processes, including limb formation, lung morphogenesis, intestinal elongation and mammary gland development. Altered WNT5A expression has been associated with a number of cancers. Interestingly, in certain types of cancers, such as hematological malignancies and colorectal carcinoma, WNT5A is inactivated and exerts a tumor suppressive function, while in other cancers, such as melanoma and gastric carcinoma, WNT5A is overexpressed and promotes tumor progression. The mechanism by which WNT5A achieves these distinct activities in cancers is poorly understood. Here, we provide evidence that the *WNT5A* gene produces two protein isoforms, WNT5A-long (WNT5A-L) and WNT5A-short (WNT5A-S). Amino-terminal sequencing and a WNT5A-L specific antibody demonstrate that the mature and secreted isoforms are distinct, with WNT5A-L carrying an additional 18 N-terminal amino acids. Biochemical analysis indicates that both purified proteins are similar with respect to their stability, hydrophobicity and WNT/β-catenin signaling activity. Nonetheless, modulation of these two *WNT5A* isoforms, either through ectopic expression or knockdown, demonstrates that they exert distinct activities in cancer cell lines: while WNT5A-L inhibits proliferation of tumor cell lines, WNT5A-S promotes their growth. Finally, we show that expression of these two WNT5A isoforms is altered in breast and cervix carcinomas, as well as in the most aggressive neuroblastoma tumors. In these cancers, WNT5A-L is frequently down-regulated, whereas WNT5A-S is found overexpressed in a significant fraction of tumors. Altogether, our study provides evidence that the distinct activities of WNT5A in cancer can be attributed to the production of two *WNT5A* isoforms.

## Introduction

The *WNT5A* gene encodes one of the 19 WNT ligand family members. Through binding to FZD (Frizzled)[1], ROR1/2 (Receptor tyrosine kinase-like Orphan Receptors)[2,3] or RYK (Receptor-like tyrosine kinase)[4] receptors and LRP5/6 (Low density lipoprotein Receptor-related Protein) co-receptors[5,6], WNT proteins modulate the canonical WNT/β-catenin signaling pathway, as well as a number of non-canonical β-catenin-independent pathways [7,8]. WNT pathways play important roles during embryogenesis and adult tissues homeostasis by regulating cell growth, proliferation, survival, adhesion and migration. Alterations in WNT signaling are frequently associated with oncogenesis [7-9]. Notably, aberrant expression of certain WNTs, inactivating mutations of the APC and AXIN tumor suppressors and oncogenic activating mutations of β-catenin (CTNNB1) have been shown to contribute to cell transformation via deregulation of genes, such as *c-MYC* and *CCND1* (cyclin D1). Epigenetic silencing of genes encoding WNT antagonists, such as the soluble decoy receptors SFRP (Secreted Frizzled Related Protein) or DKK, also leads to deregulation of the pathway and has been observed in several cancers [7]. A recent comprehensive study of colorectal cancers found that over 94% of cancers carry mutations in one or more WNT signaling components [10]. 

Among WNT signaling components implicated in oncogenesis, WNT5A is particularly interesting: it acts both as an oncoprotein and a tumor suppressor. In melanomas and gastric and pancreatic carcinomas, *WNT5A* is recurrently overexpressed and exerts a pro-oncogenic function by promoting proliferation and/or invasion and metastasis [11-16]. In contrast, *Wnt5a* heterozygous mice are predisposed to develop B cell lymphoma through loss of Wnt5a function, and *WNT5A* gene inactivation by somatic deletions or hypermethylation is frequent in human leukemia, lymphoma and colorectal carcinoma [17-20]. In addition, WNT5A inhibits proliferation of leukemia, lymphoma and colorectal carcinoma cells, demonstrating its tumor suppressive function in these cancers [17,19,20]. Finally, we and others have shown that *WNT5A* expression is down-regulated in human breast carcinomas and plays a tumor suppressor role by inhibiting proliferation and/or metastasis [21-24]. Differences in the WNT receptor repertoire, and hence in the signaling pathways triggered by WNT5A [3,12-14,17,21,22,25-27], could explain these opposing activities of WNT5A in cancer. Here, we explored an alternative mechanism by which WNT5A could exert these distinct activities, namely through the expression and production of distinct WNT5A isoforms. Specifically, we found that an amino-terminally truncated WNT5A isoform exhibits tumor-promoting activities, while the full-length WNT5A protein exhibits tumor-suppressive activities. 

## Results

### The *WNT5A* gene encodes two protein isoforms

**Figure 1:**
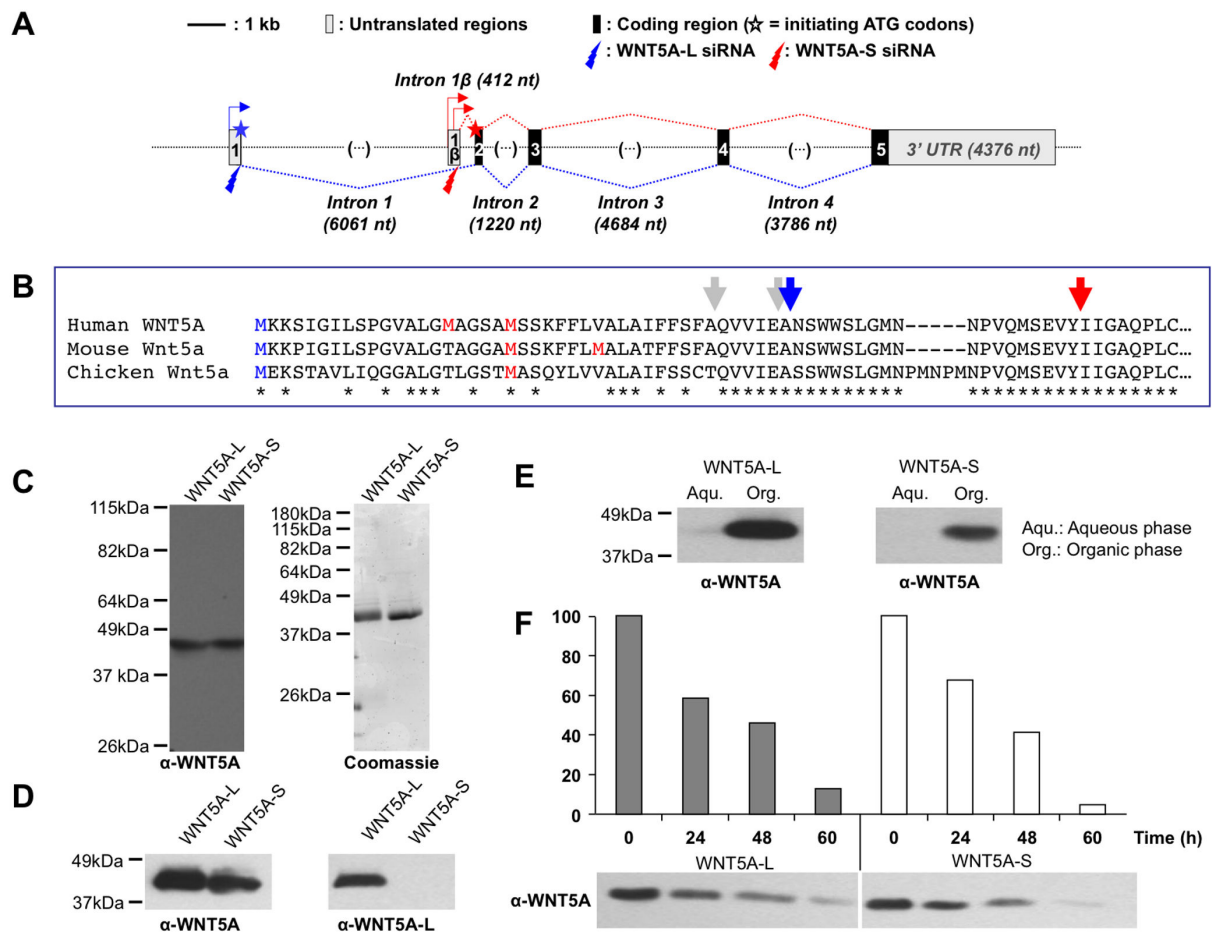
Description and biochemical chracterization of WNT5A isoforms. **A**. Structure of the human *WNT5A* gene and alternative transcripts. Numbered boxes indicate the exons, with 5’ and 3’ untranslated (UTR) regions in grey and coding regions in black. nt: nucleotides, kb: kilo base. Blue and red arrows and dotted lines respectively indicate the positions of the different transcription initiation sites in exon 1 and exon 1β and splicing of exon 1- and exon 1β-initiated transcripts. Blue and red stars indicate the translation initiation codons for the WNT5A-L and WNT5A-S isoforms located in exon 1 and exon 2, respectively. Blue and red lightning bolt arrows indicate the position of the target sequences of *WNT5A-L* and *WNT5A-S* specific short interfering RNA (siRNA). **B**. Multiple amino acid sequence alignment of the N-terminus of the WNT5A precursor proteins. The blue M denotes the most likely start codon of WNT5A-L while the red Ms denote the most likely start codons of WNT5A-S. The grey arrows indicate the positions of signal peptide cleavage sites for both isoforms as predicted by SignalP 4.1 (http://www.cbs.dtu.dk/services/SignalP/). Blue and red arrows indicate the position of the observed first amino-acids of the mature WNT5A-L and WNT5A-S proteins, respectively, and thus the position of the observed signal peptide cleavage site for each isoform. **C**. Detection of purified WNT5A isoforms. Left panel: Immuno-blot for WNT5A demonstrates that both isoforms are of similar molecular weight (^≈^43kDa). Right panel: Coomassie stained gel shows that the purified WNT5A preparations are pure with largely undetectable contaminant proteins. **D**. An anti-mouse Wnt5a antibody detects both isoforms (left panel) while an anti-rabbit WNT5A-L specific antibody (right panel) detects the WNT5A-L but not the WNT5A-S isoform. *E. Triton* X-114 phase separation demonstrates that both WNT5A isoforms partition to the hydrophobic/organic phase. WNT5A proteins were detected by immuno-blot analysis with an anti-Wnt5a antibody. **F**. Incubation of purified WNT5A isoforms at 37°C for the indicated times indicates that their *in* *vitro* stability is similar. WNT5A proteins were detected as in **E** and band intensities were quantified using ImageJ software.

To determine whether *WNT5A* encodes several protein isoforms, we analyzed sequences deposited in databases and searched for possible alternative transcripts. We found the *WNT5A* gene produces at least 3 possible *WNT5A* transcripts from alternative transcriptional start sites. One transcript initiates at exon 1 [19,20,28] and is predicted to encode a 380 amino-acid WNT5A protein precursor, from hereon referred to as WNT5A-L (Long) (Figures **1A, B**; Figures **S1A** and **S1B**). The other two transcripts initiate 718 and 578 nucleotides upstream of exon 2, in an alternative exon 1β (Figure **1A**; Figures **S1A** and **S1B**). Both exon 1β-initiated transcripts are predicted to encode a 365 or 360 amino acid (depending on use of start codon at position 16 or 21 relative to start codon of WNT5A-L) precursor protein, referred to as WNT5A-S (Short), and lack the first 15 or 20 N-terminal amino acids as compared with the WNT5A-L precursor ([29]; Figures **1A, B**; Figures **S1A** and **S1B**). 

**Figure 2:**
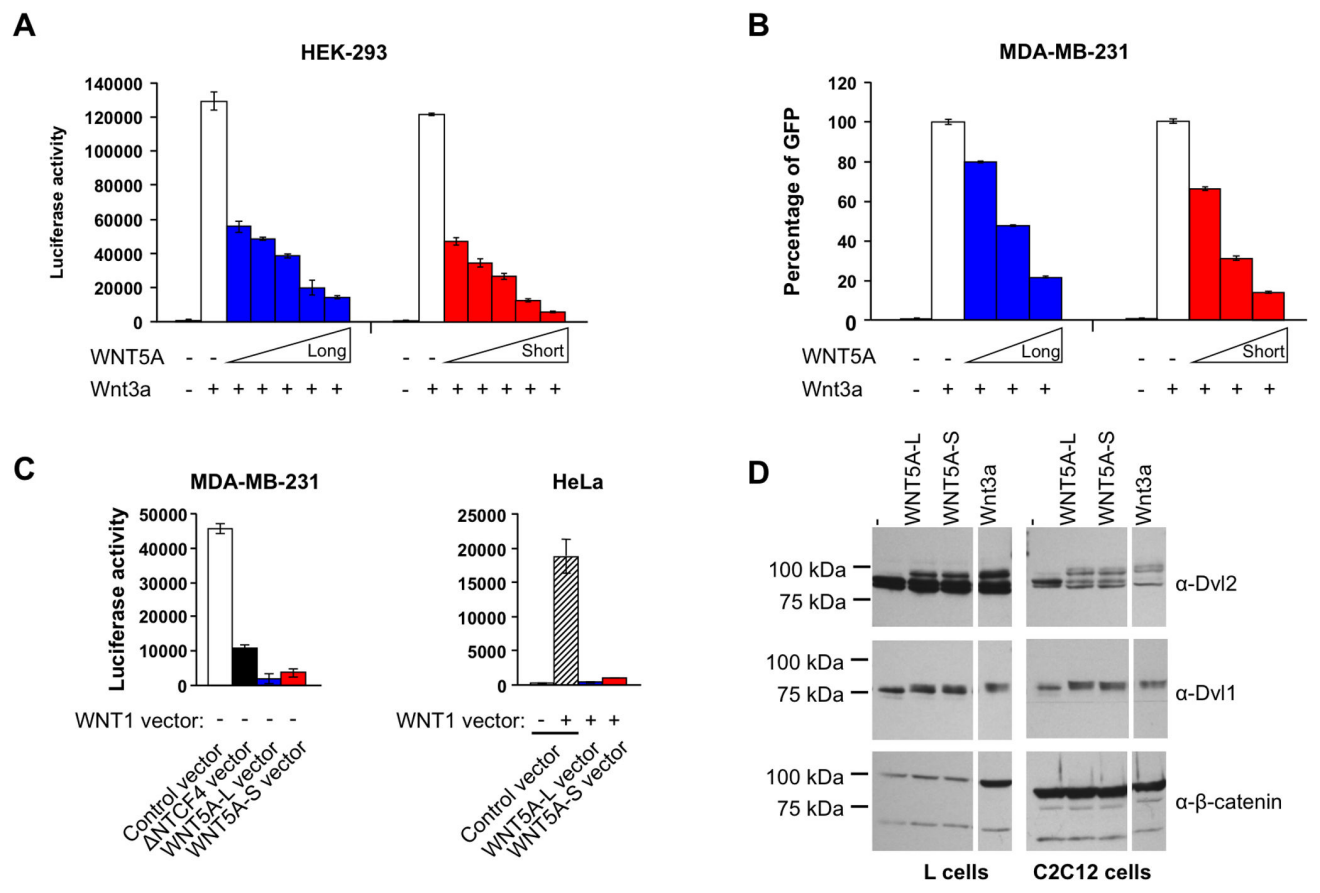
Effects of WNT5A isoforms on the β-catenin/TCF pathway and Disheveled proteins phosphorylation. Both WNT5A proteins block Wnt3a-activation of β-catenin/TCF-driven transcriptional activity in HEK 293 (TOP-Luciferase, **A**.) and MDA-MB-231 (TOP-GFP reporter, **B**.). Cells were treated with purified Wnt3a and an increasing concentration of WNT5A for 24 hours (A) or 48 hours (B). **C**. Upon transfection, both WNT5A isoforms interfere with endogenous (MDA-MB-231, left panel) and WNT1-induced (HeLa, right panel) β-catenin/TCF-driven transcription in MDA-MB-231 (left panel) and HeLa cells (right panel). Cells were transfected with control, WNT5A-L or WNT5A-S expression vectors alone, or together with a WNT1 expression vector and assayed for β-catenin/TCF-driven TOP-Luciferase reporter activity. An expression vector encoding a dominant-negative form of TCF4 (ΔNTCF4) was used to interfere with the endogenous reporter activity in MDA-MB-231 cells. **D**. Both WNT5A isoforms promote DVL phosphorylation. L cells (left panel) and C2C12 cells (right panel) were treated with WNT5A isoforms (10 nM, 2 hours) and whole cell lysates were immuno-blotted with the indicated antibodies. Both WNT5A isoforms, as well as Wnt3a (10 nM, 2 hours), led to a mobility shift of Dvl1 and Dvl2 proteins, suggesting that Dvl proteins are post-translationally modified by phosphorylation in response to both canonical (Wnt3a) and non-canonical Wnt (WNT5A) signaling. Only Wnt3a induced accumulation of the β-catenin protein. Note: β-catenin accumulation in response to Wnt3a in C2C12 cells is not detectable in these whole cell lysates because these cells contain large amounts of membrane/adherens junction associated β-catenin.

The overall exon-intron structure of the human *WNT5A* gene is highly conserved in other vertebrates, including mouse, chicken and zebrafish (Figure **S2A**). The *WNT5A* gene is split into 5 conserved exons. The length, sequence, and splice sites in codons across exons 2 - 5 are highly conserved. The untranslated region of exon 1 is more diverged, but the splice site is conserved. This exon-intron arrangement is also conserved in the closely related *WNT5B* gene, as annotated in mammalian genomes. Interestingly, the alternative exon 1β, which is embedded in the first intron, is also present in these vertebrates. In mice, exon 1β is detected in alternative Wnt5a transcripts and shares significant homology with the human sequence. In a pairwise alignment, the element encompassing the upstream regulatory region and exon 1 β are 95% conserved between human and mouse (Figure **S2B**). In birds (chicken and zebra finch), this alternative exon has not been annotated, but the presence of a highly conserved sequence with a splice donor at the 3’ end indicates that this exon 1β is present. The pairwise alignment between chicken and zebra finch of this element shows 96% conservation. Furthermore, two short sequences within these elements are highly conserved between mammals (mouse and human) and birds (chicken and zebra finch) (Figure **2B**). Fish (zebrafish and stickleback) also contain a highly conserved element within this region. Although this element has not been annotated as an exon in any database, the elevated conservation (74%) suggests the presence of an additional exon in fish (Figure **2B**). Together, the observed high degree of genomic sequence conservation suggests that alternative Wnt5a transcripts, similar to the ones described here, are present in multiple vertebrate species. Finally, alignment of the amino terminal amino acids of human, mouse and chicken Wnt5a shows that start codon M21 (position relative to start codon of WNT5A-L) is completely conserved (Figure **1B**), suggesting that WNT5A-S initiates at this position.

As is the case for most secreted proteins, Wnt precursors are cleaved at the amino terminus to remove the signal sequence thereby producing the mature polypeptide. A signal peptide cleavage prediction algorithm (*SignalP 4.1* - http://www.cbs.dtu.dk/services/SignalP/) predicts that both WNT5A precursors yield proteins of either 343 amino acids (start with QVVIEA…) or 338 amino acid (start with ANSWWS…). The latter predicted cleavage site has the highest cleavage score (Y-score of 0.625 for WNT5A-S [start at M21] and 0.246 for WNT5A-L). However, amino-terminal sequencing of mouse Wnt5a indicated that signal sequence cleavage occurred at a position 24 residues downstream from the predicted site [3]. Furthermore, since the 15 amino-acid difference in the N-terminal sequence of WNT5A-L and WNT5A-S precursors may influence the position of signal peptide cleavage, we experimentally determined the identity of the amino termini of the mature proteins. 

To identify the position of signal peptide cleavage and the first amino acid of the mature proteins, we purified both WNT5A isoforms. Using a previously developed CHO expression system [30], we independently overexpressed each WNT5A isoform and purified them from the conditioned media (CM), according to published protocols [3,31]. Both WNT5A isoforms were secreted into the CM at equal levels (data not shown), and were purified to near homogeneity (~95% pure, as assessed by Coomassie staining, Figure **1C**). Amino-terminal sequencing showed the purified WNT5A-L protein to start one amino acid downstream of the predicted signal peptide cleavage site, thus generating a 337 amino acid protein starting with the sequence NSWWS… (Figure **1B**; Figure **S1B** and **S1C**). The secreted WNT5A-S protein lacked an additional amino-terminal 18 amino acid to generate a protein of 319 amino acid starting with the sequence IIGAQ… (Figure **1B**; Figure **S1B** and **S1C**). Therefore, the two isoforms generate proteins with distinct amino terminal sequences. The WNT5A-S amino terminus matches that of mouse Wnt5a, which was previously identified by amino-terminal peptide sequencing [3]. Interestingly, the signal peptide cleavage algorithm did not predict this position as a likely site of signal sequence cleavage for either WNT5A-L or WNT5A-S (Y-score of 0.16 for WNT5A-S and 0.113 for WNT5A-L).

To confirm the distinct amino terminal composition of both mature WNT5A isoforms, we generated an antibody directed to the WNT5A-L-specific 18 amino acid (NSWWSLGMNNPVQMSEVY). In immuno-blots, the WNT5A-L-specific antibody only recognized WNT5A-L and not WNT5A-S (Figure **1D**). This demonstrates that the exon 1- and exon 1β-initiated *WNT5A* transcripts produce distinct WNT5A proteins that differ in their amino termini. The mechanism by which these two precursors are differentially processed to yield distinct mature proteins is currently unclear, however, we speculate that differences in the amino termini influence the choice of the processing machinery to cleave the proteins at distinct positions. Further studies are needed to address these questions.

WNT proteins are highly hydrophobic, a property imparted by the covalent attachment of at least one lipid molecule to a conserved residue [31,32]. This modification is important for proper processing and secretion [32,33]. In addition, as revealed by the Wnt-Fzd co-crystal structure, the lipid moiety is critical for receptor binding [34]. To address whether the two WNT5A isoforms differed in their hydrophobic properties, and hence in their lipid modifications, we used the phase separation property of the detergent Triton X-114. We observed no difference between the two isoforms, as they both partitioned equally to the organic phase (Figure **1E**), thus suggesting that both isoforms are similarly modified. To test whether the two isoforms differed in their stability, we incubated each protein at 37°C for up to 60 hours. Immuno-blot detection indicated that both proteins degraded at equal rates (Figure **1F**). Therefore, the two WNT5A isoforms behave similarly with respect to their *in vitro* stability and hydrophobicity. It is, however, possible that each isoform has distinct biochemical properties in an *in vivo* setting, a possibility we have not addressed.

### Effects of WNT5A-L and WNT5A-S isoforms on signaling pathways

Several WNT signaling pathways have been described and proposed. The most commonly studied and best-established pathway, often referred to as the canonical WNT pathway, involves β-catenin as a central mediator. Other non-canonical pathways, which act independently of β-catenin, are poorly understood. At the very minimum, these two pathways are thought to share the following components: WNT ligands, FZD receptors and Dishevelled (DVL) signal transducers. Although WNT5A is predominantly associated with non-canonical WNT signaling, it has also been found to activate canonical β-catenin signaling in certain conditions [3]. We investigated if the WNT5A isoforms differentially impact the β-catenin/TCF signaling pathway. To this end, we generated two cell lines, HEK 293 and MDA-MB-231, carrying a WNT/β-catenin specific reporter system. This reporter system consists of a WNT responsive element comprised of 7 multimerized TCF binding sites (referred to as TOP for TCF Optimal Promoter [35,36]), which regulates the expression of a reporter gene, either Luciferase for HEK 293 cells (HEK293-TOP-Luciferase, Figure **2A**; Figure **S3A**) or GFP for MDA-MB-231 cells (MDA-MB-231-TOP-GFP, Figure **2B**; Figure **S3B**). These reporter cells are potently activated by Wnt3a (Figure **2A and B**), a WNT protein commonly used to stimulate the canonical signaling pathway. In contrast, neither WNT5A isoform activated or consistently modulated the basal activity of these reporters (Figure **S3A** and **S3B**), indicating that these cellular systems do not provide the appropriate milieu to connect WNT5A signaling to the canonical β-catenin pathway. 

Sensitive and robust cell-based assays for non-canonical WNT signaling are scarce. However, non-canonical WNT signaling has been shown to antagonize WNT/β-catenin signaling, an activity that can be assayed using the TOP-reporter system [3]. We found that both WNT5A isoforms inhibit WNT3A-induced reporter activation in a dose-dependent manner (Figure **2 A and B**). These results show that, despite the difference in their amino-termini, both WNT5A proteins potently antagonize the WNT/β-catenin signaling. Similar inhibitory effects of WNT5A-L and WNT5A-S isoforms on the WNT/β-catenin-driven reporter were observed when cells were transfected with expression vectors (Figure **2C**) rather than treated with purified WNT5A-L and WNT5A-S proteins. Therefore, in these assays, the WNT5A isoforms exhibit similar biological activities when delivered as purified proteins or transfected genes. 

WNT signaling, canonical and non-canonical, leads to the hyperphosphorylation of Dvl proteins, signaling molecules that act immediately downstream of FZD receptors. This post-translational modification of Dvl proteins can be readily detected by an electrophoretic mobility shift [37-39]. We found that treatment of two cell lines (mouse L cells and the mouse myoblast C2C12 cell line) with either WNT5A isoform, as well as WNT3A, induces a mobility shift of Dvl proteins, as detected by immuno-blotting for Dvl1 and Dvl2 (Figure **2D**). However, in agreement with the distinct activities of WNT3A and WNT5A isoforms on WNT/β-catenin signaling (Figures **2A, 2B**; Figure **S3A** **, S3B**), only Wnt3a, but not WNT5A isoforms, induced accumulation of the β-catenin protein, as evidenced in L cells. This β-catenin protein accumulation is not apparent in C2C12 cells, which already express high levels of β-catenin (Figure **2D**). Taken together, our data indicate that the two WNT5A isoforms exhibit identical biological activities in the established cell-based WNT signaling assays. 

### Expression of WNT5A isoforms in cancer cell lines and normal tissues

**Figure 3:**
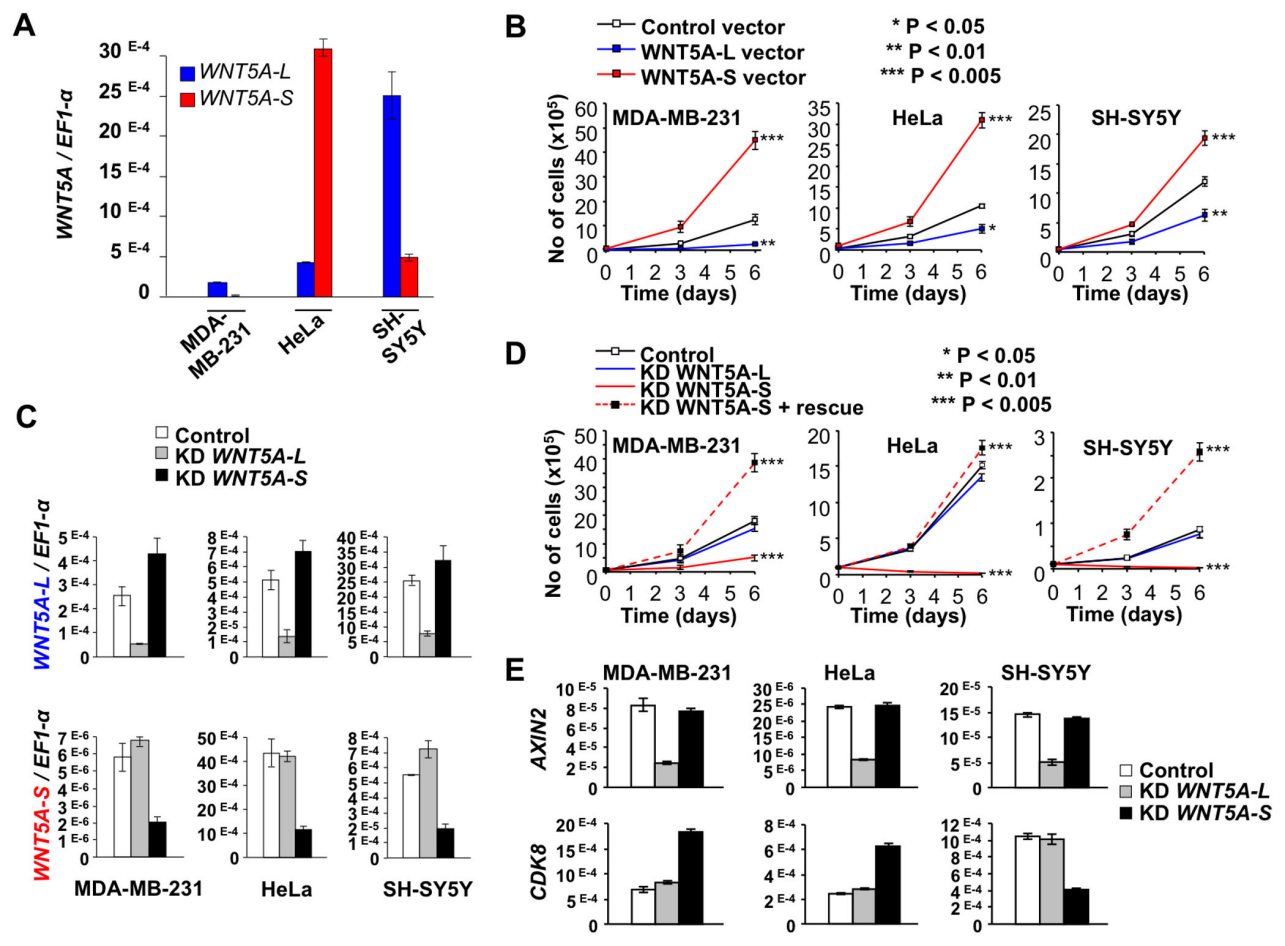
Effects of WNT5A isoform expression on cancer cell line proliferation. **A**. WNT5A isoform expression in three cancer cell lines. Transcript levels of *WNT5A* isoforms were determined in MDA-MB-231 (breast carcinoma), HeLa (cervix carcinoma), and SH-SY5Y (neuroblastoma) by qRT-PCR and normalized by *EF1-α* mRNA (normalization by *GAPDH* mRNA and *18S* rRNA produced similar results). Mean ratios (*WNT5A* isoforms/ *EF1-α*) ± SEM from independent measurements are shown. **B**. Effects of ectopic expression of WNT5A-L and WNT5A-S isoforms on proliferation. The indicated cell lines were transduced with expression vectors encoding WNT5A-L (blue line), WNT5A-S (red line) or no WNT (Black line) and cell numbers were determined at 3 and 6 days. WNT5A-S increases while WNT5A-L decreases proliferative rates. Data (mean ± SEM from triplicate determinations) from a representative experiment are shown. Each experiment was performed at least three independent times. **C**. Isoform specific siRNAs reduce expression of each WNT5A isoform. The efficiency of WNT5A-L and WNT5A-S isoform knockdown (KD) was evaluated in MDA-MB-231, HeLa and SH-SY5Y transfected with control siRNA and *WNT5A* isoform-specific siRNA (KD). *WNT5A* isoforms transcript levels were measured by quantitative RT-PCR (qRT-PCR) and normalized by *EF1-α* mRNA (normalization by *GAPDH* mRNA and *18S* rRNA produced similar results). **D**. Effects of siRNA-mediated knockdown (KD) of WNT5A-L and WNT5A-S isoforms on proliferation. Cells were transfected with siRNAs specific to WNT5A-L (blue line) or WNT5A-S (red line) and cell numbers were determined at 3 and 6 days. A scrambled siRNA served as control (black line). Co-transduction of cells with a WNT5A-S expression vector rescues the effect of the WNT5A-S-specific siRNA (dashed red line). Data (mean ± SEM from triplicate determinations) from a representative experiment are shown. Each experiment was performed at least three independent times. * *P* < 0.05; ** *P* < 0.01; *** *P* < 0.005; **** *P* < 0.001. **E**. Differential regulation of *AXIN2* and *CDK8* expression by isoform-specific knockdown of WNT5A. Transcript levels of *AXIN2* and *CDK8* were determined by quantitative RT-PCR analysis (normalized by *EF1-α* mRNA) in control MDA-MB-231 (breast carcinoma), HeLa (cervix carcinoma) and SH-SY5Y (neuroblastoma) cells, and in response to siRNA-mediated knock-down (KD) of WNT5A-L or WNT5A-S. Mean ratios ± SEM from independent measurements are shown.

Previous studies of WNT5A expression have not discriminated between the two isoforms described here. Therefore, we measured their transcript levels in cancer cell lines and in normal tissues by performing quantitative RT-PCR (qRT-PCR) with isoform-specific primers (see Table **S1** and Figure **S1**). This analysis showed that expression of these isoforms is highly variable between individual cancer cell lines. For example, while MDA-MB-231 (derived from a breast carcinoma) and SH-SY5Y (derived from a neuroblastoma) express predominantly *WNT5A-L*, HeLa cells (derived from a cervix carcinoma) express predominantly *WNT5A-S* (Figure **3A**). Other cell lines, including MCF-7 (derived from a breast carcinoma) or IMR-32 (derived from a neuroblastoma) express extremely low levels of either WNT5A isoform (Figure **S4A**). Although less quantitative, gel analysis of end point RT-PCR products obtained from representative cell lines confirmed the qRT-PCR data (Figure **S4A**). We next analyzed the transcript levels of WNT5A isoforms in normal adult tissues. We detected *WNT5A-L* expression in nearly all tissues, except adipose tissue. In contrast, we detected *WNT5A-S* expression only in placenta, and, to a lesser extent, in trachea and small intestine (Figure **S4B**).

### WNT5A-S promotes and WNT5A-L suppresses growth of cancer cell lines

To study the functions of the WNT5A isoforms, we manipulated their expression in several cell lines, including MDA-MB-231, HeLa and SH-SY5Y, and analyzed the phenotypic consequences (Figure **3B-D**; Figure **S5**). Since signaling activities of purified WNT proteins are short-lived [30] and biological assays generally require long periods of stimulation, we utilized transfection of *WNT* genes rather than application of purified proteins. Ectopic expression of WNT5A-L substantially reduced cell numbers in these cell lines (Figure **3B**). No evidence of increased cell death was observed (data not shown), indicating that WNT5A-L acts to inhibit cell proliferation. In contrast, ectopic expression of WNT5A-S promoted proliferation of MDA-MB-231, HeLa and SH-SY5Y cells (Figure **3B**).

Using isoform-specific siRNAs (see Figure **S1**; Table **S2**), we disrupted expression of each WNT5A isoform (Figure **3C**). The siRNAs were able to specifically knockdown their targets up to 80% as determined by qRT-PCR (Figure **3C**). Importantly, each WNT5A isoform was selectively inhibited by the corresponding siRNA without affecting levels of the other isoform. Consistent with the proliferation promoting effects of WNT5A-S ectopic expression, knockdown of WNT5A-S reduced cell numbers (Figure **3D**). Even in cells expressing low WNT5A-S levels, such as MDA-MB-231 (Figure **3A**), knockdown of the WNT5A-S isoform produced a strong inhibitory effect (Figure **3D**). The cell inhibition resulting from WNT5A-S siRNA was rescued in all tested cell lines by co-transduction with a WNT5A-S vector devoid of the siRNA target sequence (Figure **3D**, KD WNT5A-S + rescue). These rescuing data demonstrate that the effects of this siRNA are due to WNT5A-S knock-down rather than non-specific off-target effects. Furthermore, WNT5A-S knockdown increased cell death in the three cell lines (Figure **S5A**) and induced caspase activity in MDA-MB-231 and HeLa (Figure **S5B**). However, the ≥3-fold reduction in cell numbers (Figure **3D**) exceeded the observed modest increase in cell death (Figure **S5A**), suggesting that WNT5A-S knockdown mainly impaired proliferation. In contrast, knockdown of WNT5A-L had no effect on cell proliferation (Figure **3D**), cell death or caspase activity in the 3 cell lines (Figure **3D**; Figure **S5A, B**). Taken together, these experiments demonstrate that endogenously and ectopically expressed WNT5A isoforms exert distinct effects in breast, cervix and neuroblastoma tumor cell lines, with WNT5A-S promoting and WNT5A-L inhibiting cell proliferation. Since we did not observe distinct signaling activities for WNT5A-L and WNT5A-S in WNT/β-catenin and DVL phosphorylation assays (Figure **2**), we speculate that these growth promoting and inhibiting activities of WNT5A are independent of the canonical WNT signaling pathway. 

In an attempt to identify a mechanism by which WNT5A isoforms exert differential effects on proliferation, we screened the expression of several known WNT regulated genes. We found that expression of two genes, *AXIN2* and *CDK8*, both of which have been implicated in various aspects of WNT signaling [7,40,41], were differentially regulated by isoform-specific knockdown of WNT5A. Knockdown of WNT5A-L, but not WNT5A-S, led to a decrease in *AXIN2* expression in 3 tested cell lines (MDA-MB-231, HeLa and SH-SY5Y, Figure **3E**). In contrast, knockdown of WNT5A-S, but not WNT5A-L, led to an increase in *CDK8* expression in 2 out of 3 cell lines (MDA-MB-231 and HeLa) and a decrease in SH-SY5Y (Figure **3E**). This data offers a potential molecular link between the observed differential effects of WNT5A isoforms on proliferation and gene regulation and indicates that each WNT5A isoform impinges on gene expression in a distinct manner. Further exhaustive studies, such as transcriptome analysis, will be needed to identify and define pathways that mediate these differential effects. 

### Down-regulation of WNT5A-L and overexpression of the WNT5A-S isoform in cancers

**Figure 4:**
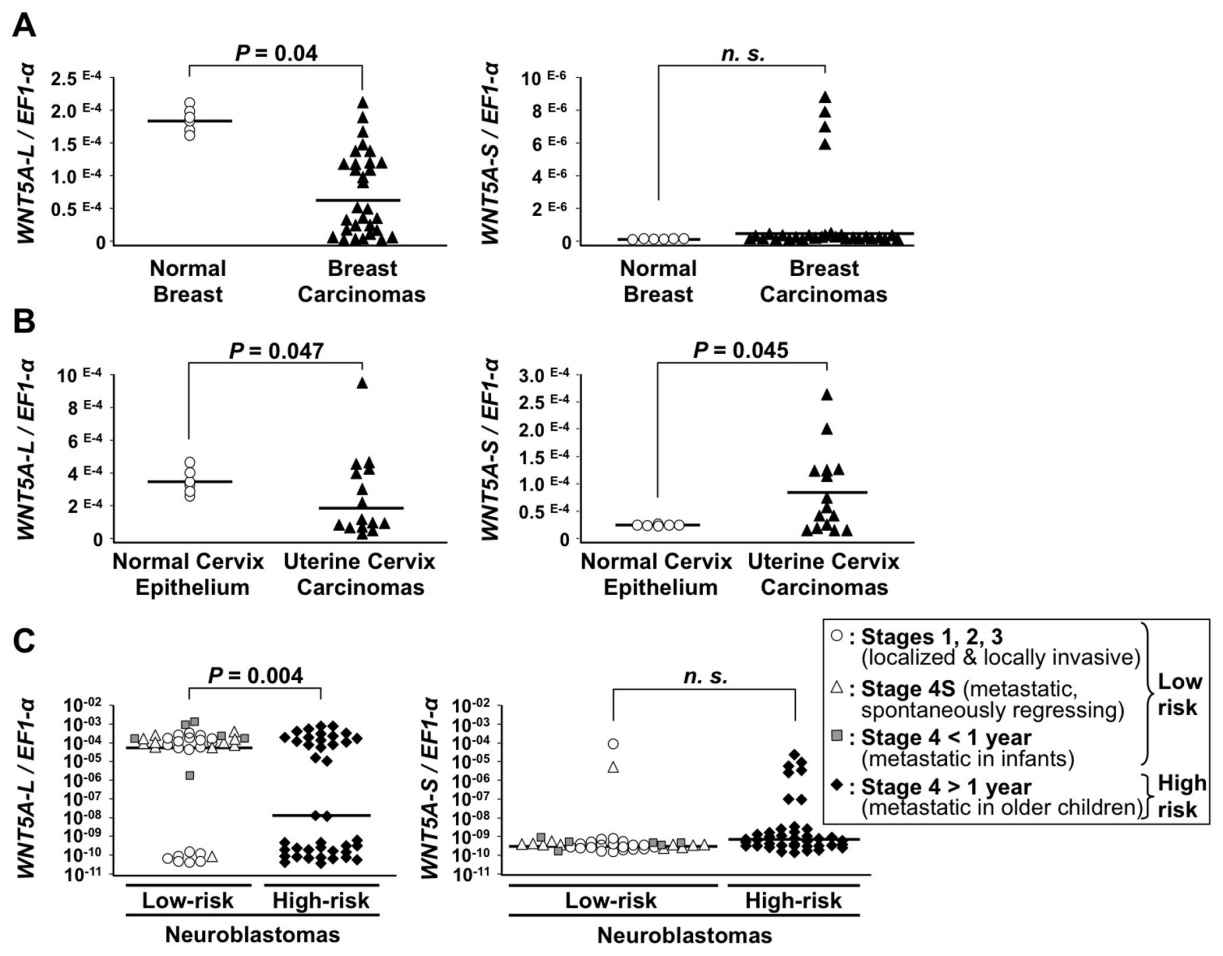
Altered expression of WNT5A isoforms in cancers. Transcript levels of *WNT5A-L* (left panels) and *WNT5A-S* (right panels) isoforms were measured in different types of cancers by qRT-PCR and normalized by *EF1-α* mRNA (normalization by *GAPDH* mRNA and *18S* rRNA produced similar results). Symbols represent mean ratios (*WNT5A* isoforms/ *EF1-α*) from at least two independent measurements for each sample. Horizontal bars correspond to the mean expression value of *WNT5A* isoforms in each subgroup of samples. For breast (**A**) and uterine cervix (**B**), expression in carcinomas was compared to that in normal tissues. For neuroblastomas (NB, C), expression was compared between low risk tumors with good prognosis (Stages 1, 2, 3: localized or locally invasive NB; Stage 4S: spontaneously regressing metastatic NB; Stage 4 < 1 year, metastatic NB of infants) and high risk tumors with poor prognosis (Stage 4 > 1 year: metastatic NB of older children). n.s. = not significant.

To determine whether the respective pro- and anti-proliferative functions of the WNT5A-S and WNT5A-L isoforms are relevant to oncogenesis, we analyzed their expression levels by qRT-PCR in primary tumor samples from breast and cervix carcinomas and neuroblastomas. As compared to normal breast tissues, *WNT5A-L* was down-regulated ≥3-fold in 46% (14 of 30) of breast carcinomas (*P*= 0.04) (Figure **4A**). *WNT5A-S* was undetectable in normal breast tissue and overexpressed in a small subset of breast cancers (4 of 30, 13%, n. s.) (Figure **4A**). In normal uterine cervix epithelium, both *WNT5A-L* and *WNT5A-S* were expressed, *WNT5A-L* being the predominant isoform (Figure **4B**). In comparison with normal cervix, *WNT5A-L* was down-regulated ≥3-fold in 53% (8 of 15) of cervix carcinomas (*P*= 0.047). In contrast, *WNT5A-S* was up-regulated ≥4-fold in 40% (6 of 15) of these tumors (*P*= 0.045) (Figure **4B**). Neuroblastomas, pediatric cancers of the sympathetic nervous system, can be classified into low and high risk tumors with 90% and 30% of survival, respectively. Half of the high risk neuroblastomas are fatal within 2 years from diagnosis [42]. High risk neuroblastomas showed low *WNT5A-L* expression more frequently than low risk tumors (55% -22 of 40- versus 21% -8 of 37-, *P*= 0.004), thus suggesting that WNT5A-L down-regulation correlates with high risk neuroblastoma. Furthermore, elevated *WNT5A-S* expression was associated more frequently with high risk than with low risk neuroblastomas (17% -7 of 40- versus 5% -2 of 37-, n. s.) (Figure **4C**). In our cohort, 5% (2 of 40) of patients with low risk neuroblastomas died over a 10-year follow-up period, in contrast with 70% (half of them within 2 years from diagnosis) in the high risk group. In this latter group of the most aggressive tumors, low expression of *WNT5A-L* or high expression of *WNT5A-S* were both associated with shorter survival (mean= 26.05 versus 37.1 months for *WNT5A-L* and 27.56 versus 36.2 months for *WNT5A-S*). However, this association was not statistically significant. High risk neuroblastomas carry several genetic alterations, each of high prognostic value [42]. Therefore, only analysis of larger cohorts of patients will determine whether the expression status of *WNT5A-L* or *WNT5A-S* isoforms is of further relevance to clinical outcome. 

As *WNT5A* inactivation is frequent in hematological malignancies [17,19], we also analyzed the expression status of its isoforms in these cancers and corresponding normal tissue. Normal blood cells exclusively express the WNT5A-L isoform, which was strongly down-regulated in most of the myeloid and B-cell lymphoid leukemia as well as T-cell lymphoma samples (Figure **S6**). The WNT5A-S isoform was only marginally expressed in one B-cell leukemia and one T-cell lymphoma, indicating that this isoform is not significantly implicated in hematological cancers. These results are consistent with previous observations [17,19], however, these previous studies did not distinguish between the two WNT5A isoforms. Thus, in these selected human tumors, the WNT5A-L and WNT5A-S isoforms behave as tumor suppressive and oncogenic factors, respectively. 

## Discussion

Our work reveals that the *WNT5A* gene encodes two distinct transcripts expressed from alternative transcriptional start sites, producing messenger RNAs with distinct translational start sites. These two types of transcripts lead to production of two distinct WNT5A proteins, a long 337 amino acids isoform (WNT5A-L) and a short 319 amino acids isoform (WNT5A-S). Amino-terminal peptide sequencing and a WNT5A-L specific antibody confirmed the existence of two distinct WNT5A proteins. A recent study analyzed the transcriptional regulation of the *WNT5A* gene and, consistent with our studies, identified two isoforms that differed in their transcriptional start sites [43]. While this previous study elegantly elucidated the upstream regulatory elements controlling expression of each isoform, our current study demonstrates that two distinct WNT5A proteins (post-signal sequence cleavage) are produced and that the two isoforms exert opposing activities in cancer cell lines. 

Alternative promoter usage to generate multiple transcripts is common, including among *WNT* genes. In addition to *WNT5A*, *WNT16* and *WNT2B* also produce alternative transcripts that are differentially expressed in human tissues and cancers [44-46]. Therefore, through use of alternative transcriptional start sites, the resulting Wnt family of proteins may be significantly more complex and diverse than expected for 19 genes. 

Importantly, we showed, through ectopic expression and knockdown studies, that the WNT5A-S isoform promoted cell proliferation while the WNT5A-L isoform inhibited cell proliferation. Furthermore, we found that WNT5A-S expression was elevated in a fraction of breast and cervix carcinoma as well as neuroblastoma tumors, whereas WNT5A-L expression was down-regulated in these cancers. These findings offer a novel mechanism by which WNT5A can exert either oncogenic or tumor suppressive activities. Our observations indicate that the distinct functions of WNT5A in cancer are not only due to differences in cellular context (e.g. expression of receptors and other signal transducers). Instead, we propose that two distinct WNT5A isoforms, WNT5A-L and WNT5A-S, possess intrinsically different activities and behave as tumor suppressor and oncogene, respectively. The mechanisms by which these differential effects are achieved are currently unknown. Along these lines, we found that expression of two genes, *AXIN2* and *CDK8*, is differentially regulated by these two isoforms. Future studies are needed to further define how gene regulation is differentially affected by these two WNT5A isoforms.

The *WNT5A* gene was previously found silenced in colorectal and hematological cancers due to hypermethylation of a CpG island encompassing exon 1 [19,20], the initiation site of the WNT5A-L isoform. Here, we have shown that normal colon and normal blood cells exclusively express WNT5A-L and that this isoform is frequently silenced in hematological cancers. Together, these observations indicate that the WNT5A-L isoform mediates the tumor suppressive functions of WNT5A in colorectal and hematological cancers. It will be of high interest to determine which isoform of WNT5A exerts oncogenic functions in melanomas and gastric and pancreatic carcinomas [11-16], and monitoring the respective expression levels of WNT5A-L and WNT5A-S may be a useful cancer biomarker. The roles of WNT5A during development [25-28,47-50] also warrant evaluation of the functions of the isoforms in normal ontogenesis and their potential implications in developmental disorders. 

Finally, we have shown that the distinct effects of WNT5A-L and WNT5A-S isoforms on proliferation of several cell lines are not due to differential effects on the WNT/β-catenin pathway. The WNT5A-L and WNT5A-S isoforms may therefore regulate distinct alternative signaling pathways that mediate their respective tumor suppressive and oncogenic functions. Characterization of these pathways, including the identification of possible receptors, signal transduction components and target gene expression, will be necessary to gain a better understanding of the roles of these isoforms in carcinogenesis and may enable the identification of novel therapeutic targets. Indeed, treatments that could restore the effects of the tumor suppressive WNT5A isoform or block that of the oncogenic variant may confer clinical benefit in cancers in which alterations of these isoforms are implicated. 

## Materials and Methods

### Ethics Statement

According to the French Public Health and Bioethical Law, our study was considered by our Research Direction Lawyer (Direction de la Recherche Clinique, Centre Hospitalier Universitaire de Bordeaux, France) as a non interventional study without need for ethics committee approval (Article L1121-1 and Article R1121-3). The need for written informed consent from the participants was not waived, and, according to that law, all patients (or their legal tutors for pediatric tumors, herein neuroblastomas) received oral and written information concerning research on their material with possibility to refuse the use of their clinical data and biological samples. None of the patients whose material was analyzed in this study refused such use and all patients signed an informed consent form. The present study complied with the Declaration of Helsinki Principles and was approved by the medical and ethics committee of the Centre Hospitalier Universitaire de Bordeaux (Bordeaux, France) and Institut de Cancérologie Gustave Roussy (Villejuif, France).

### Patient tissues and cell collection

Normal and cancerous tissues and cells were collected and stored frozen at -80°C prior to nucleic acid extraction. Samples with estimated cancer cells content of more than 70% on the basis of cyto-pathological review were retained for analysis. RNA from a series of normal adult tissues were purchased from Ambion.

### Plasmids and antibodies

The sequences encoding WNT5A-Long and WNT5A-Short isoforms were separately amplified by polymerase chain reaction (PCR) using cDNA from HeLa cells as a template. PCR products were cloned in the pCDNA3.1(+)Neo expression vector (Invitrogen) and constructs were verified by sequencing. For the WNT5A-S producing CHO line we engineered a construct lacking the sequence for the WNT5A-L specific peptide (NSWWSLGMNNPVQMSEVY). Generation of CHO cell lines have been described elsewhere [30]. The SuperTOPFLASH luciferase reporter was kindly provided by the laboratory of Dr. R. Moon. The TOP-GFP construct was obtained from Addgene (Plasmid #24305, described in Fuerer and Nusse 2010 PLoS One[51]). The WNT5A antibody has been described elsewhere [3]. The WNT5A-L specific antibody was generated by immunizing two rabbits with the peptide specific to the long isoform (NSWWSLGMNNPVQMSEVY). Immunizations, serum collections and exsanguination were performed by Epitomics, Inc. Antisera were tested by immuno-blotting of WNT5A-L and WNT5A-S. The antisera were affinity purified against the peptide (NSWWSLGMNNPVQMSEVY) immobilized to Sepharose beads. The Dvl2 antibody was generated by immunizing two rabbits with a GST fusion protein to the 86 carboxy terminal amino acids of mouse Dvl2. Antisera were affinity purified against the immunogen. The rabbit anti-Dvl1 antibody was provided by Epitomics, and the mouse anti-β-catenin antibody was purchased from Sigma-Aldrich.

### Cell culture

MDA-MB-231, MCF-7, T-47D, SK-Br-3, HeLa, SH-SY5Y, SK-N-AS, IMR-32, HEK-293, L cells and C2C12, all obtained from the ATCC (American Tissue Culture Collection), IGR-N-91[52], IGR-NB8 [53], LAN-1 [54], LAN-5 (Life Technologies) and CLB-Pe [55] cell lines were grown in DMEM supplemented with 10% fetal bovine serum. BT-474 cells (ATCC), were grown in RPMI 1640 supplemented with 10% fetal bovine serum. CHO cells (ATCC) were cultured in DMEM supplemented with 10% bovine serum, 1% non essential amino acids (Gibco), 400µg/ml of G418, 10ng/ml Doxycycline and 2ug/ml Blasticidin. 

### RNA expression analysis

RNA was isolated from freshly frozen tumor or cell samples and matched normal counterparts using the "RNeasy mini" kits (QIAGEN). For gene expression analysis, cDNA synthesis was performed from total RNA using the SuperScript II reverse transcriptase kit (Invitrogen). For quantitative reverse transcription-PCR (qRT–PCR), we used the SYBR Green detection system (Applied Biosystems) for housekeeping genes and a TaqMan probe (Applied Biosystems) for *WNT5A* transcripts. Sequences of qPCR primers and probe are given in Table S1. 

### Transfections and WNT activity assays

Short interfering RNA (siRNA, sequences in Table S2) were transfected using Oligofectamine (Invitrogen). Plasmids were transfected using Lipofectamine LTX (Invitrogen). Co-transfection of siRNA and plasmids were performed using Lipofectamine 2000 (Invitrogen). For RNA analyses, cells were collected after 3 days. For proliferation and viability assays, cells were harvested after 3 and 6 days. In case of transfection with plasmids, cells were selected for 6 days using 1 mg/ml of G418. Resistant cells were replated in equal number and grown for up to 6 days before counting. For luciferase reporter assays, stable HEK293 transfectants carrying a TOP-Luciferase reporter element (Addgene plasmid 12456) were plated in 96 well plates and collected after 24h of WNT treatment and assayed using the Luciferase Assay kit (Promega) on a luminometer (Perkin Elmer Envision). For GFP reporter assays, stable MDA-MB-231 transfectants carrying a TOP-GFP reporter element (Addgene plasmid 35489) were collected after 48h of WNT treatment and regulation of TOP-GFP was monitored by flow cytometry (BD FACS Canto). For the Dvl phosphorylation assay, L1 and C2C12 cells were grown to ~80% confluence, treated with WNT proteins at 10 μM for 2 hours, lysed on ice in 1% Triton X-100, 150 mM NaCl, 50 mM Tris-Cl, pH7.5, and protease inhibitors Leupeptin (1 μg/ml), Aprotinin (10 μg/ml), and Pefabloc (1 mM) and the phosphatase inhibitor β-Glycerophosphate (10 mM). 20 μg total soluble whole cell lysate was resolved by SDS-polyacrylamide gel electrophoresis, transferred to nitrocellulose and immuno-blotted with the indicated antibodies.

### Proliferation and viability

Cells transfected with plasmids and/or siRNA were harvested by trypsin treatment after 3 and 6 days and assessed for proliferation and viability using Trypan Blue exclusion and counting with a ViCell counter (Beckman).

### Statistical analysis

The Student’s t-test was used to compare the expression levels of WNT5A isoforms in two groups of samples and the proliferation of control and genetically engineered cells. All graphs represent mean values ± s.e.m. Clinical outcome was analyzed in neuroblastoma patients as a function of WNT5A isoforms expression levels. Kaplan-Meier curves were used to assess overall survival, and differences between groups were compared by the log-rank test. * : P < 0.05; ** : P < 0.01; *** : P < 0.005; **** : P < 0.001 (when P values are not indicated on the figure).

### WNT5A protein expression, purification and characterization

WNT5A isoforms were expressed in CHO cells [30] and conditioned media (CM) were collected the day after confluence was reached. WNT proteins were purified from 6 liters of CHO CM. CM was complemented with 1% Triton X-100 (v/v), 20 mM Tris-Cl pH 7.5 and 0.01% NaN_3_. The purification method consists of four consecutive steps performed on an Äkta Purifier (GE Healthcare) using the method previously described [56]. WNT yields were determined by Coomassie staining of SDS-polyacrylamide gels. Yields for WNT5A isoforms averaged at 40µg per liter of CM. Stability of WNT5A isoforms was tested by spiking HEK 293 growth media with equal amounts of purified WNT5A isoforms, and incubating at 37°C for the indicated time points. Hydrophobicity of WNT5A proteins was assessed by Triton X-114 phase separation. 500µl of CM was mixed with 500µl Triton buffer (2.2% Triton X-114; 300mM NaCl; 20mM Tris-HCl, pH7.5). The mixture was incubated 5min on ice, then 4min at 31°C and subsequently centrifuged at 2000xg for 3min at 31°C. The two phases (top = aqueous, bottom = hydrophobic/organic) were separated and assayed for protein by immuno-blot.

## Supporting Information

Figure S1 **Sequence of the human WNT5A gene, transcripts and protein.** **A**. Nucleotide sequence of the WNT5A gene. Blue and red arrows indicate the positions of the different transcriptional initiation sites in exon 1 and exon 1β. 5’ and 3’ UTR regions are in purple characters and the promoter and 3’ flanking regions are in green. The predicted start codons (ATG) of the WNT5A-L and WNT5A-S protein precursors located in exons 1 and 2, respectively, are framed in red. The stop codon, common to both isoforms and located in exon 5, is framed in blue. The coding region in exons 1, 2, 3, 4 and 5 is in bold black characters. Intronic sequences are in blue lower case italics. Intron 1 (6061 nucleotides, nt) is spliced from the mature WNT5A-L transcript, which initiates in exon 1. Exon 1β, which is located within intron 1 splices to exon 2 and produces the WNT5A-S transcript (see text and Figure 1A), is in blue capitals with purple shaded frame. The 412 nt region spliced from mature exon 1β-initiated transcripts is delineated by red brackets and is in blue lower case italics. Sequences complementary to the qPCR TaqMan probe and reverse primer, both common to all WNT5A transcripts, and isoform-specific forward primers are underlined by arrows. Sequences complementary to the oligonucleotide used for primer extension by K.G. Danielson et al. [29] and sequences targeted by isoform-selective short interfering RNA (siRNA) are also underlined. **B**. Complete complementary DNA (cDNA) and peptide sequences of WNT5A-L (Left) and WNT5A-S (Right) isoforms. Nucleotide sequences of exon 1 and exon 1β are indicated in black, and sequences of exons 2, 3, 4 and 5 are alternate with red and blue to indicate the boundaries of each exon. Coding sequences are underlined and amino acids corresponding to each codon are indicated below the cDNA sequences. Black numbers on the left margin indicate amino acid positions. Amino acid residues encoded by codons that straddle a splice junction are marked in grey. The most likely start codons of the WNT5A-L and WNT5A-S protein precursors, M1, M16 and M21 (M = Methionine, numbers refer to positions in the WNT5A-L isoform precursor), with average prediction scores of 0.9145, 0.7286 and 0.629 (determined by two distinct algorithms: *TIS* *Miner* - http://dnafsminer.bic.nus.edu.sg/Tis.html- and ATG ^*Pr*^ - http://atgpr.dbcls.jp/ -), respectively, located in exons 1 and 2, are framed in red. Other potential but less likely start codons (M51 and M57 with *ATG* ^*Pr*^ and TIS Miner prediction scores of 0.594 and 0.576, respectively) are framed by black dotted lines. Notably, *ATG* ^*Pr*^ predicts only M1 for WNT5A-L and only M16 for WNT5A-S. The positions of the observed first amino acids (as determined by amino-terminal sequencing), and likely position of the signal peptide cleavage sites, are indicated by arrows (blue for WNT5A-L and red for WNT5A-S). After signal peptide cleavage in their N-Terminal regions, WNT5A-L and WNT5A-S begin at Asparagine (N) 44 and Isoleucine (I) 62, respectively (numbers refer to amino acid positions relative to M1 in the WNT5A-L isoform precursor). The first 2 amino acids (MK) of the WNT5A-L isoform precursor , indicated by black letters and underlined, are encoded in exon 1. The STOP codon, common to both isoforms and located in exon 5, is framed in blue. **C**. Amino terminal sequencing of WNT5A proteins. Purified WNT5A proteins were resolved by SDS- polyacrylamide gel electrophoresis and Coomassie stained proteins were excised and submitted for amino-terminal peptide sequencing (Stanford School of Medicine PAN Facility). Numbers next to the single letter amino acid code indicate the yield in pmoles. Letters in parenthesis indicate secondary amino acids detected in each cycle of the Edman degradation sequencing reactions.
(TIF)

Figure S2 **Genomic conservation of *WNT5A* gene.** **A**. The overall exon-intron structure of the Wnt5a gene is highly conserved in vertebrates. The top two stick diagrams represent the two WNT5A transcripts analyzed in this study. Grey boxes indicate untranslated regions. Blue and red boxes indicate the coding region of WNT5A-L and WNT5A-S, respectively. The black line denotes the position of the start codon. The lower three black stick diagrams represent the overall exon-intron structure of human, chimpanzee and mouse (top), chicken and zebra finch (middle) and zebrafish (bottom). Length and sequence of exons 2 and 5 are highly conserved in all vertebrates, while length of exon 1 is variable. The alternative exon 1b is depicted as an open box (black for human, chimp and mouse, blue for chicken and zebra finch, and orange for zebrafish). The length of introns is not depicted to scale. **B**. Multiple sequence analysis of exon 1β shows high degree of conservation amongst several vertebrate species. Conserved sequence elements identified in the first intron of the *Wnt5a* gene were aligned pairwise to show the high degree of conservation between human (hs) and mouse (mm), chicken (gg) and zebra finch (tg), and zebrafish (dr) and stickleback (ga). Shown for each alignment are the upstream region (ups) and exon 1b, either annotated (human and mouse) or predicted (chicken, zebra finch, zebrafish and stickleback). Shown in yellow are highly conserved regions shared between human, mouse, chicken and zebra finch. Shown in teal are the conserved canonical splice donor sites.
(TIF)

Figure S3 **WNT5A isoforms do not activate WNT/β-catenin specific reporters.** β-catenin/TCF-driven transcriptional activity is monitored in HEK 293 using a TOP-Luciferase reporter (**A**) and in MDA-MB-231 using a TOP-GFP reporter (**B**). Cells were treated for 24 hours (Luciferase, A) or 48 hours (GFP) with varying amounts of purified WNT5A or with Wnt3a. In contrast to Wnt3a (right panels), neither WNT5A isoforms activate the reporter activity (left panels).
(TIF)

Figure S4 **Expression levels of *WNT5A* isoform transcripts in cancer cell lines and in normal human adult tissues.** Transcript levels of WNT5A isoforms were determined by quantitative RT-PCR (qRT-PCR) with isoform-specific primers in breast, cervix and neuroblastoma tumor cell lines (A) and normal tissues (**B**), and normalized by *EF1-α* mRNA (normalization by *GAPDH* mRNA and *18S* rRNA produced similar results). Mean ratios (*WNT5A* isoforms/ *EF1-α*) ± SEM from 3 independent measurements are shown. **A**. The highest levels of *WNT5A* transcripts were detected in HeLa (cervix carcinoma) and SH-SY5Y and SK-N-AS (neuroblastoma) cell lines. Lower *WNT5A* levels were found in MDA-MB-231 and SK-Br-3 (breast carcinoma) and IGR-N-91 and LAN-1 (neuroblastoma) cell lines. Other cell lines showed weak or undetectable *WNT5A* expression. Agarose (4%) gel analysis of duplex RT-PCR performed with primers for both *WNT5A* isoforms (35 cycles), normalized by RT-PCR of *EF1-α* mRNA (25 cycles), confirmed the qRT-PCR data. **B**. With the exception of adipose tissue, which does not express detectable *WNT5A*, all tested normal adult tissues, including germinal tissues, express the *WNT5A-L* isoform, at variable levels. Expression of *WNT5A-S* is more restricted and was detected only at low levels in the small intestine, at moderate levels in the trachea and at high levels (higher than the *WNT5A-L* isoform) in the placenta. WNT5A-S was not detected in all other tested adult tissues.
(TIF)

Figure S5 **Effect of WNT5A isoforms on proliferation and viability of carcinoma and neuroblastoma cells.** **A**. Impact of siRNA-mediated knock-down (KD) of WNT5A-L and WNT5A-S on proliferation and viability of carcinoma (MDA-MB-231 –breast- and HeLa –cervix-) and neuroblastoma (SH-SY5Y) cells. Control and isoform-specific *WNT5A* siRNA (KD) transfected cells were harvested after 6 days (adherent as well as non-adherent if present), incubated with trypan blue dye and counted using ViCell (Beckman). Bar graphs depict the numbers of viable and dead cells (Mean ± SEM from triplicate determinations of a representative experiment are shown; each experiment was performed at least three times independently). The efficiency of WNT5A-L and WNT5A-S isoforms knockdown was determined by qRT-PCR (**see** Figure 3C). **B**. Effects of knock-down of WNT5A isoforms on Caspase activity in carcinomas and neuroblastoma cells. Cells (adherent and non-adherent if present) transfected with control and isoform-specific *WNT5A* siRNA (KD) were assessed for Caspase activity using a luminescent assay to measure the activity of Caspase 3 and 7 (Caspase-Glo^®^ 3/7 Assay, *Promega*). Control and WNT5A-L and WNT5A-S knocked-down cells were mixed with a substrate, which becomes luminescent upon Caspase 3 or 7 cleavage. The resulting luminescence was measured using a luminometer (Berthold). Means ± SEM from independent experiments are shown and reveal that knockdown of WNT5A-S, but not of WNT5A-L, causes a significant increase of Caspase 3/7 activity in MDA-MB-231 (breast) and HeLa (cervix) carcinoma cells. No effect was observed in SH-SY5Y neuroblastoma cells.
(TIF)

Figure S6 **Expression of *WNT5A* isoforms in human hematological malignancies.** *WNT5A-L* and *WNT5A-S* transcripts levels were determined by qRT-PCR and normalized by *EF1α* mRNA (*18S* rRNA produced similar results). Mean ratios (*WNT5A* isoforms/ *EF1-α*) ± SEM from 3 independent measurements are shown. Normal blood cells (BM, bone marrow; PBMC, peripheral blood mononuclear cells) exclusively express the *WNT5A-L* isoform. Consistent with reported homozygous deletions [17] or exon 1 hypermethylation-associated silencing of the *WNT5A* gene in these malignancies [19], we observed that expression of the exon 1-initiated *WNT5A-L* isoform is strongly down-regulated or silenced in the vast majority of childhood acute myeloid (AML) and B-cells lymphoblastic leukemias (B-ALL), as well as in adult T-cells lymphomas (TCL), but rarely in childhood acute T-cells lymphoblastic leukemias (T-ALL). The *WNT5A-S* isoform was only marginally expressed in these samples and was detected at low levels in only one B-ALL and one TCL sample.
(TIF)

Table S1 **Sequences of primers used for quantitative RT-PCR.**
(DOCX)

Table S2 **Sequences of siRNAs.**
(DOCX)
