# Supplementary figures and images for: WNT5A Encodes Two Isoforms with Distinct Functions in Cancers
Source: PLoS One. 2013 Nov 18;8(11):e80526. doi: 10.1371/journal.pone.0080526 (PMC3832467; doi:10.1371/journal.pone.0080526)

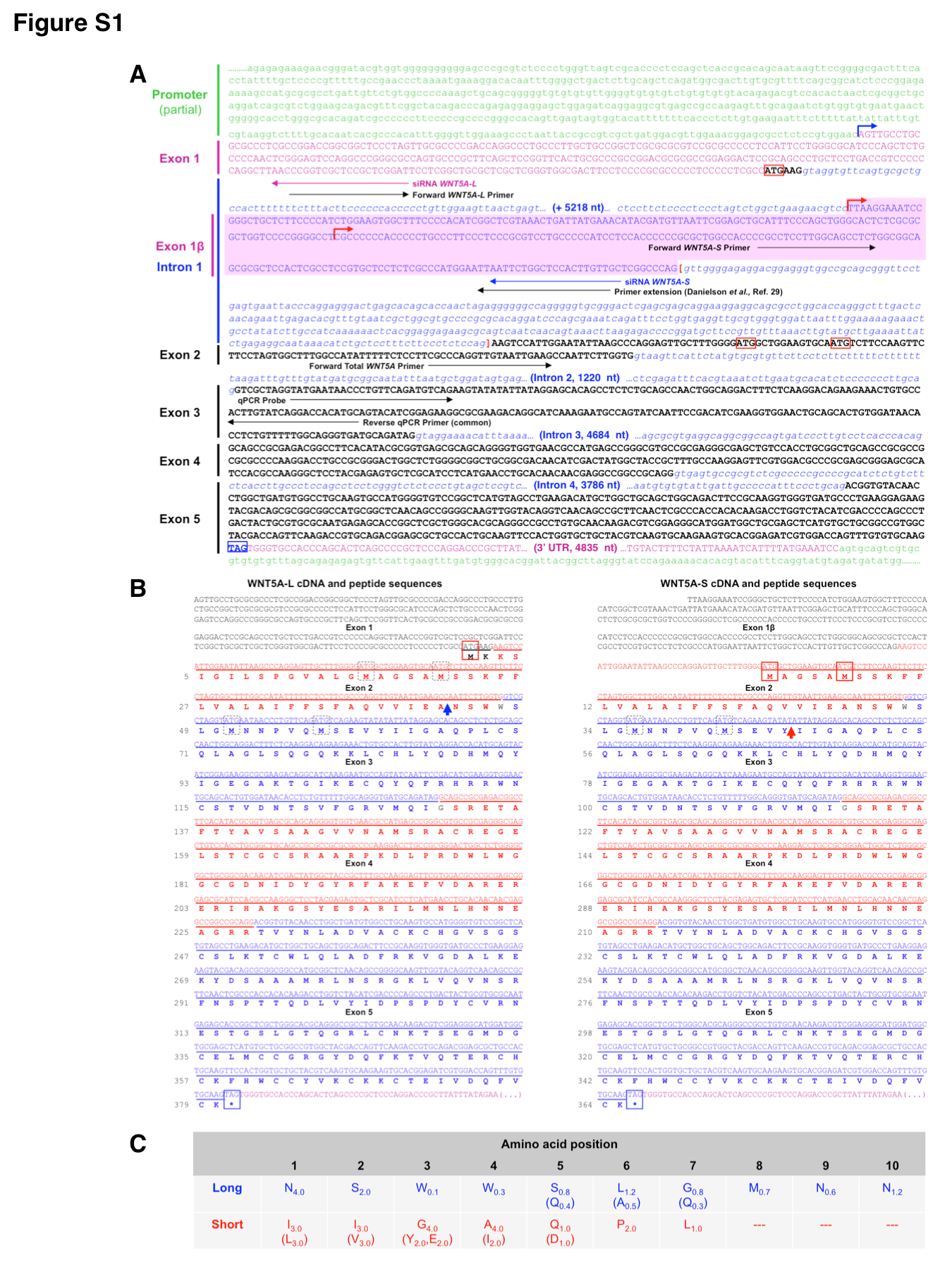

Supplement: Figure S1 — Sequence of the human WNT5A gene, transcripts and protein. A. Nucleotide sequence of the WNT5A gene. Blue and red arrows indicate the positions of the different transcriptional initiation sites in exon 1 and exon 1β. 5’ and 3’ UTR regions are in purple characters and the promoter and 3’ flanking regions are in green. The predicted start codons (ATG) of the WNT5A-L and WNT5A-S protein precursors located in exons 1 and 2, respectively, are framed in red. The stop codon, common to both isoforms and located in exon 5, is framed in blue. The coding region in exons 1, 2, 3, 4 and 5 is in bold black characters. Intronic sequences are in blue lower case italics. Intron 1 (6061 nucleotides, nt) is spliced from the mature WNT5A-L transcript, which initiates in exon 1. Exon 1β, which is located within intron 1 splices to exon 2 and produces the WNT5A-S transcript (see text and Figure 1A), is in blue capitals with purple shaded frame. The 412 nt region spliced from mature exon 1β-initiated transcripts is delineated by red brackets and is in blue lower case italics. Sequences complementary to the qPCR TaqMan probe and reverse primer, both common to all WNT5A transcripts, and isoform-specific forward primers are underlined by arrows. Sequences complementary to the oligonucleotide used for primer extension by K.G. Danielson et al. [29] and sequences targeted by isoform-selective short interfering RNA (siRNA) are also underlined. B. Complete complementary DNA (cDNA) and peptide sequences of WNT5A-L (Left) and WNT5A-S (Right) isoforms. Nucleotide sequences of exon 1 and exon 1β are indicated in black, and sequences of exons 2, 3, 4 and 5 are alternate with red and blue to indicate the boundaries of each exon. Coding sequences are underlined and amino acids corresponding to each codon are indicated below the cDNA sequences. Black numbers on the left margin indicate amino acid positions. Amino acid residues encoded by codons that straddle a splice junction are marked in grey. The most [file pone.0080526.s001.tif]

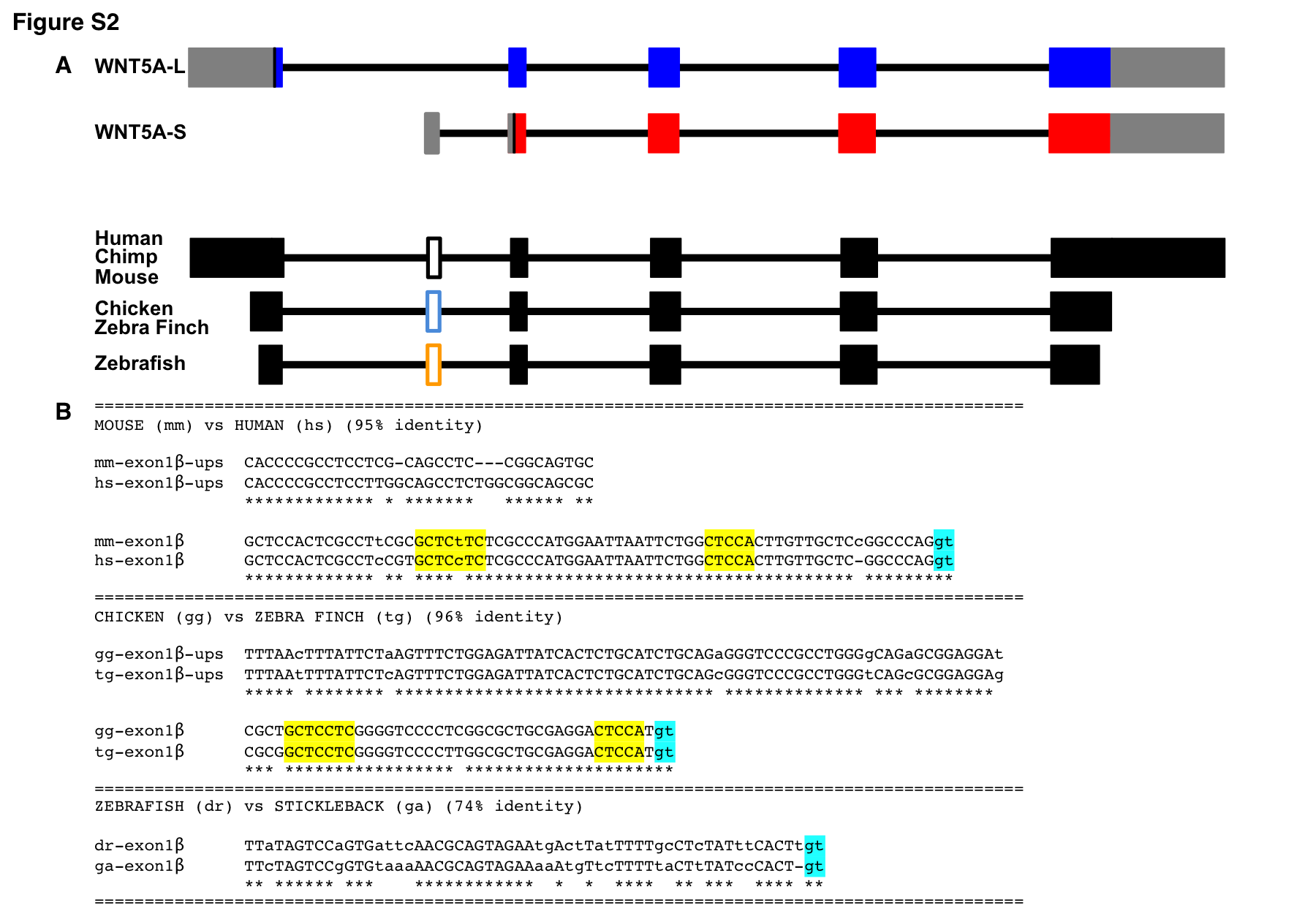

Supplement: Figure S2 — Genomic conservation of WNT5A gene. A. The overall exon-intron structure of the Wnt5a gene is highly conserved in vertebrates. The top two stick diagrams represent the two WNT5A transcripts analyzed in this study. Grey boxes indicate untranslated regions. Blue and red boxes indicate the coding region of WNT5A-L and WNT5A-S, respectively. The black line denotes the position of the start codon. The lower three black stick diagrams represent the overall exon-intron structure of human, chimpanzee and mouse (top), chicken and zebra finch (middle) and zebrafish (bottom). Length and sequence of exons 2 and 5 are highly conserved in all vertebrates, while length of exon 1 is variable. The alternative exon 1b is depicted as an open box (black for human, chimp and mouse, blue for chicken and zebra finch, and orange for zebrafish). The length of introns is not depicted to scale. B. Multiple sequence analysis of exon 1β shows high degree of conservation amongst several vertebrate species. Conserved sequence elements identified in the first intron of the Wnt5a gene were aligned pairwise to show the high degree of conservation between human (hs) and mouse (mm), chicken (gg) and zebra finch (tg), and zebrafish (dr) and stickleback (ga). Shown for each alignment are the upstream region (ups) and exon 1b, either annotated (human and mouse) or predicted (chicken, zebra finch, zebrafish and stickleback). Shown in yellow are highly conserved regions shared between human, mouse, chicken and zebra finch. Shown in teal are the conserved canonical splice donor sites. (TIF) [file pone.0080526.s002.tif]

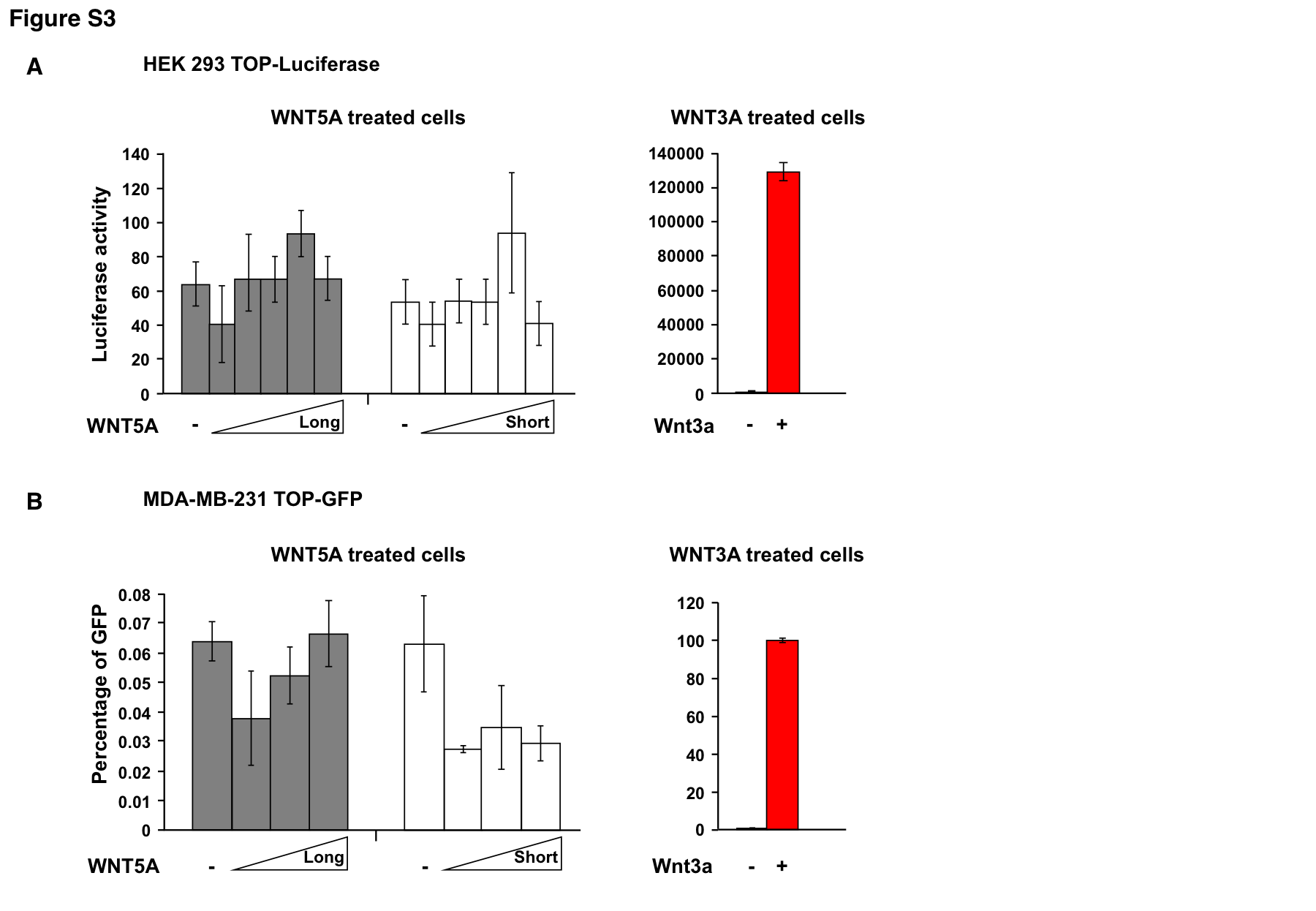

Supplement: Figure S3 — WNT5A isoforms do not activate WNT/β-catenin specific reporters. β-catenin/TCF-driven transcriptional activity is monitored in HEK 293 using a TOP-Luciferase reporter (A) and in MDA-MB-231 using a TOP-GFP reporter (B). Cells were treated for 24 hours (Luciferase, A) or 48 hours (GFP) with varying amounts of purified WNT5A or with Wnt3a. In contrast to Wnt3a (right panels), neither WNT5A isoforms activate the reporter activity (left panels). (TIF) [file pone.0080526.s003.tif]

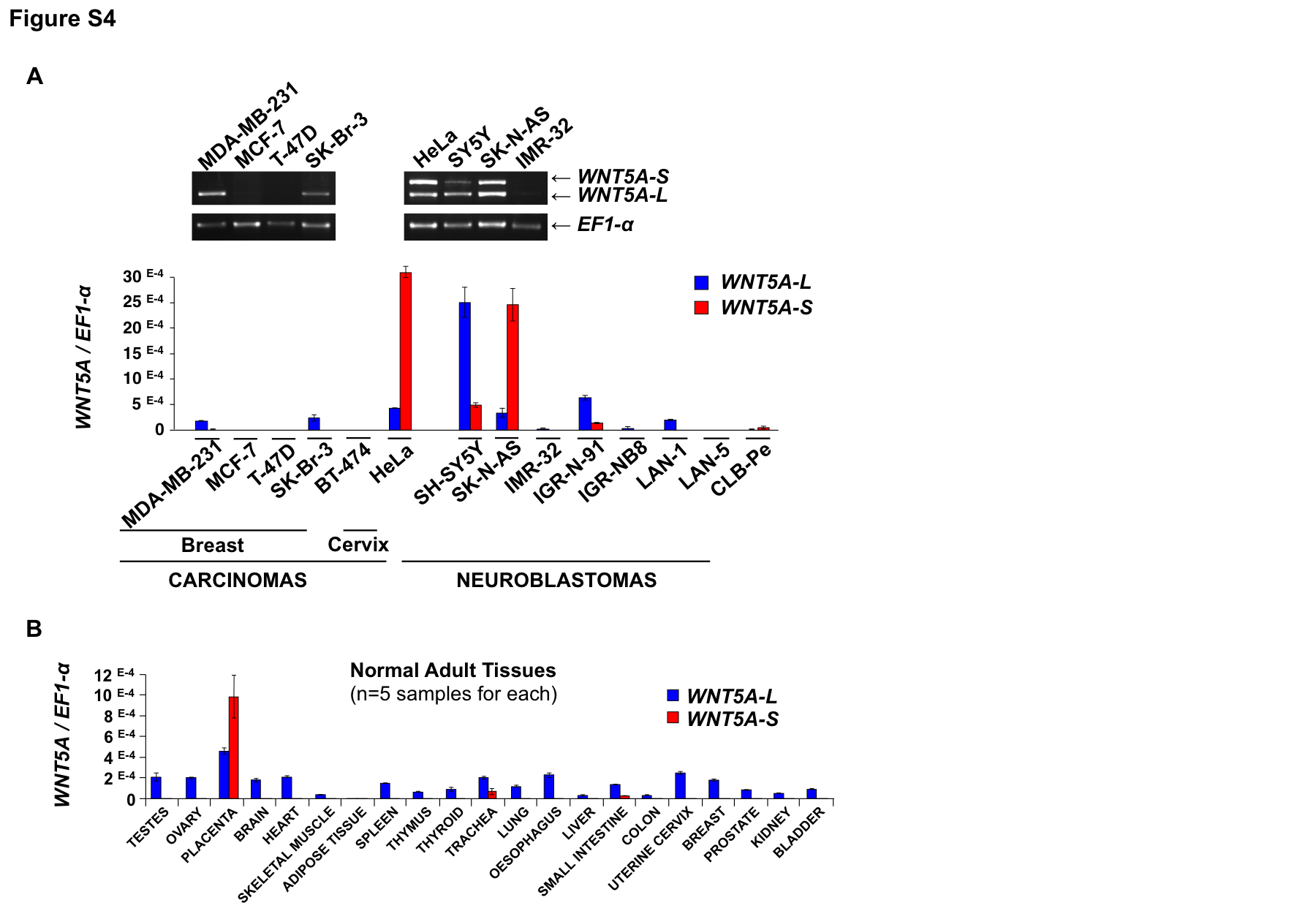

Supplement: Figure S4 — Expression levels of WNT5A isoform transcripts in cancer cell lines and in normal human adult tissues. Transcript levels of WNT5A isoforms were determined by quantitative RT-PCR (qRT-PCR) with isoform-specific primers in breast, cervix and neuroblastoma tumor cell lines (A) and normal tissues (B), and normalized by EF1-α mRNA (normalization by GAPDH mRNA and 18S rRNA produced similar results). Mean ratios (WNT5A isoforms/ EF1-α) ± SEM from 3 independent measurements are shown. A. The highest levels of WNT5A transcripts were detected in HeLa (cervix carcinoma) and SH-SY5Y and SK-N-AS (neuroblastoma) cell lines. Lower WNT5A levels were found in MDA-MB-231 and SK-Br-3 (breast carcinoma) and IGR-N-91 and LAN-1 (neuroblastoma) cell lines. Other cell lines showed weak or undetectable WNT5A expression. Agarose (4%) gel analysis of duplex RT-PCR performed with primers for both WNT5A isoforms (35 cycles), normalized by RT-PCR of EF1-α mRNA (25 cycles), confirmed the qRT-PCR data. B. With the exception of adipose tissue, which does not express detectable WNT5A, all tested normal adult tissues, including germinal tissues, express the WNT5A-L isoform, at variable levels. Expression of WNT5A-S is more restricted and was detected only at low levels in the small intestine, at moderate levels in the trachea and at high levels (higher than the WNT5A-L isoform) in the placenta. WNT5A-S was not detected in all other tested adult tissues. (TIF) [file pone.0080526.s004.tif]

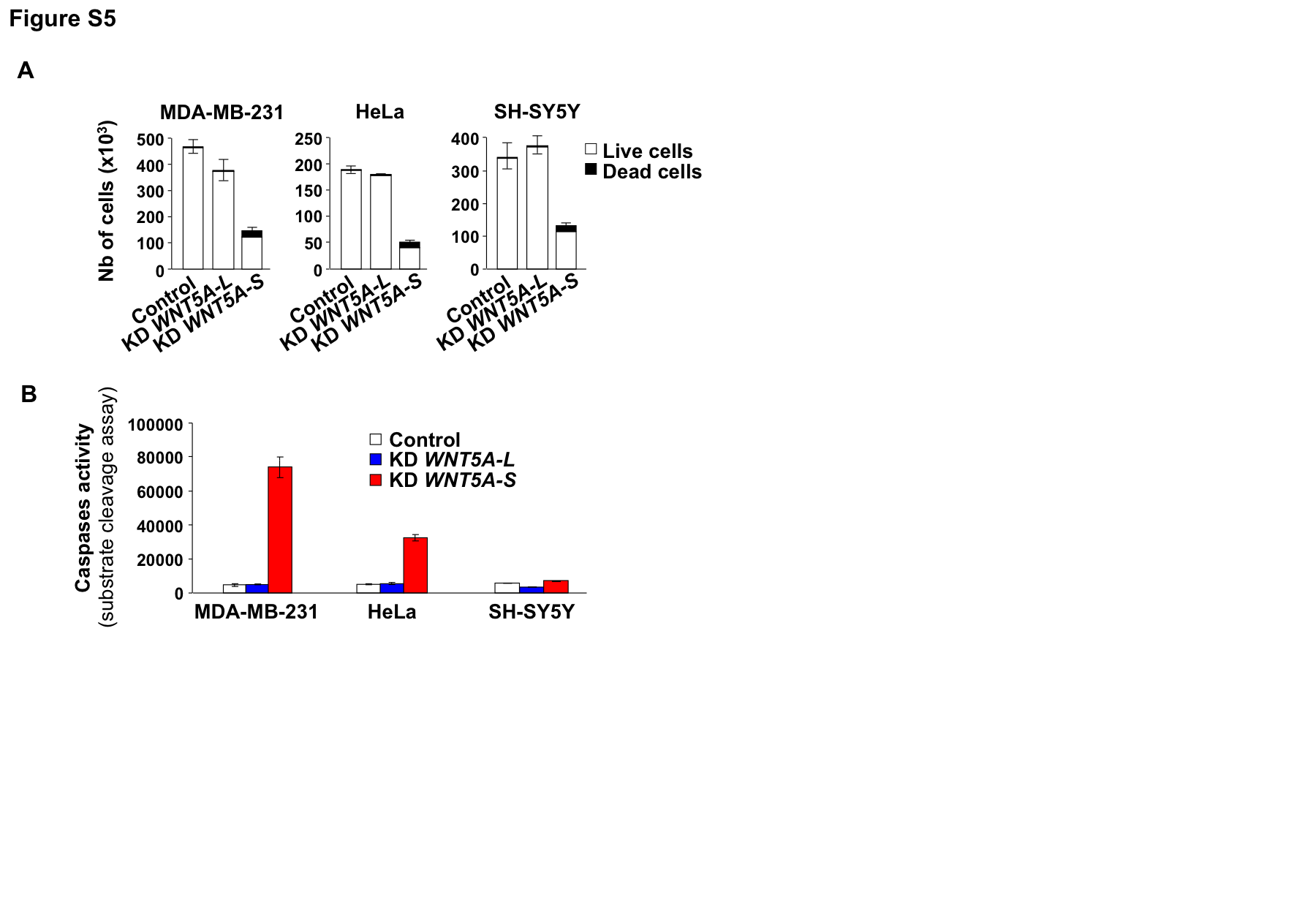

Supplement: Figure S5 — Effect of WNT5A isoforms on proliferation and viability of carcinoma and neuroblastoma cells. A. Impact of siRNA-mediated knock-down (KD) of WNT5A-L and WNT5A-S on proliferation and viability of carcinoma (MDA-MB-231 –breast- and HeLa –cervix-) and neuroblastoma (SH-SY5Y) cells. Control and isoform-specific WNT5A siRNA (KD) transfected cells were harvested after 6 days (adherent as well as non-adherent if present), incubated with trypan blue dye and counted using ViCell (Beckman). Bar graphs depict the numbers of viable and dead cells (Mean ± SEM from triplicate determinations of a representative experiment are shown; each experiment was performed at least three times independently). The efficiency of WNT5A-L and WNT5A-S isoforms knockdown was determined by qRT-PCR (see Figure 3C). B. Effects of knock-down of WNT5A isoforms on Caspase activity in carcinomas and neuroblastoma cells. Cells (adherent and non-adherent if present) transfected with control and isoform-specific WNT5A siRNA (KD) were assessed for Caspase activity using a luminescent assay to measure the activity of Caspase 3 and 7 (Caspase-Glo® 3/7 Assay, Promega). Control and WNT5A-L and WNT5A-S knocked-down cells were mixed with a substrate, which becomes luminescent upon Caspase 3 or 7 cleavage. The resulting luminescence was measured using a luminometer (Berthold). Means ± SEM from independent experiments are shown and reveal that knockdown of WNT5A-S, but not of WNT5A-L, causes a significant increase of Caspase 3/7 activity in MDA-MB-231 (breast) and HeLa (cervix) carcinoma cells. No effect was observed in SH-SY5Y neuroblastoma cells. (TIF) [file pone.0080526.s005.tif]

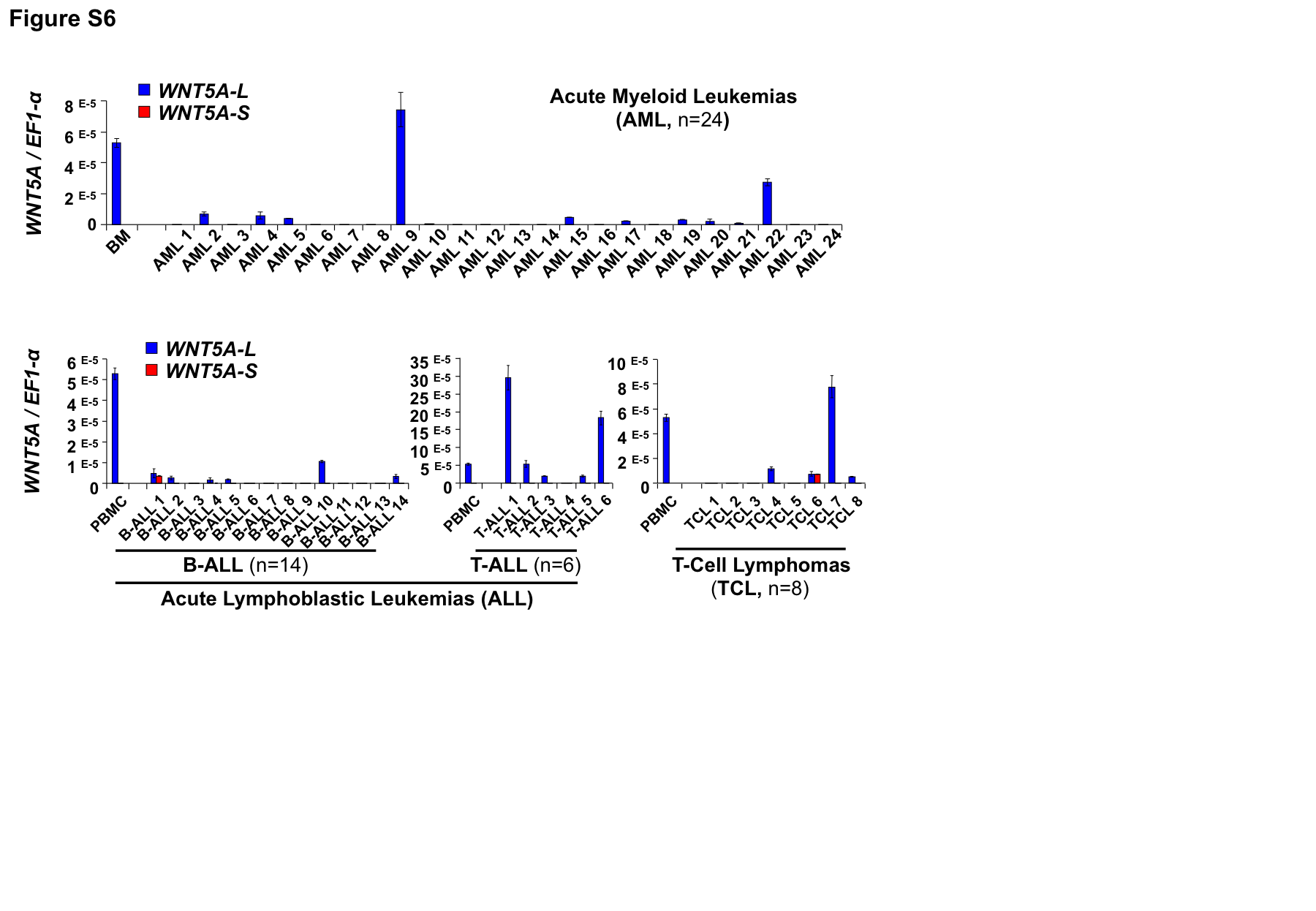

Supplement: Figure S6 — Expression of WNT5A isoforms in human hematological malignancies. WNT5A-L and WNT5A-S transcripts levels were determined by qRT-PCR and normalized by EF1α mRNA (18S rRNA produced similar results). Mean ratios (WNT5A isoforms/ EF1-α) ± SEM from 3 independent measurements are shown. Normal blood cells (BM, bone marrow; PBMC, peripheral blood mononuclear cells) exclusively express the WNT5A-L isoform. Consistent with reported homozygous deletions [17] or exon 1 hypermethylation-associated silencing of the WNT5A gene in these malignancies [19], we observed that expression of the exon 1-initiated WNT5A-L isoform is strongly down-regulated or silenced in the vast majority of childhood acute myeloid (AML) and B-cells lymphoblastic leukemias (B-ALL), as well as in adult T-cells lymphomas (TCL), but rarely in childhood acute T-cells lymphoblastic leukemias (T-ALL). The WNT5A-S isoform was only marginally expressed in these samples and was detected at low levels in only one B-ALL and one TCL sample. (TIF) [file pone.0080526.s006.tif]
