# Supplementary material for: WNT5A Encodes Two Isoforms with Distinct Functions in Cancers
Source: PLoS One. 2013 Nov 18;8(11):e80526. doi: 10.1371/journal.pone.0080526 (PMC3832467; doi:10.1371/journal.pone.0080526)
Supplement: Table S1 — Sequences of primers used for quantitative RT-PCR. (DOCX) [file pone.0080526.s007.docx]

**Table S1: Sequences of primers used for quantitative RT-PCR.**

***WNT5A (wingless-related MMTV integration site 5A):***

Forward primer, *WNT5A* transcripts (both isoforms, exon 2): 5‘-CGCCCAGGTTGTAATTGAAG-3‘

Forward primer, *WNT5A-L* isoform (exon 1): 5‘-CCGGTCGCTCCGCTCGGAT-3‘

Forward primer, *WNT5A-S* isoform (exon 1β): 5‘-CGCCTCCTTGGCAGCCTCT-3‘

Reverse primer, *WNT5A* transcripts (both isoforms, exon 3): 5‘-GCATGTGGTCCTGATACAAGT-3‘

qPCR probe, *WNT5A* (both isoforms, exon 3): 5’-TATGAATAACCCTGTTCAGATGTCAG-3’

Product sizes:

- Amplicon with primers in exons 2 and 3, common to all *WNT5A* transcripts: 164 base pairs
- Amplicon with primers in exons 1 and 3, specific of *WNT5A-L* transcripts: 347 base pairs
- Amplicon with primers in exons 1β and 3, specific of *WNT5A-S* transcripts: 363 base pairs

***AXIN2:***

Forward primer: 5‘-ATTCGGCCACTGTTCAGACG -3‘

Reverse primer: 5‘-GACAACCAACTCACTGGCCTG -3‘

Product size: 122 base pairs

***CDK8 (Cyclin-dependent kinase 8):***

Forward primer: 5‘-AGGCACTTATGGTCACGTCTA-3‘

Reverse primer: 5‘-CTTCCTATCAGCATGAGACAG -3‘

Product size: 190 base pairs

**Housekeeping genes serving as controls for normalization:**

***18S rRNA (18S ribosomal RNA):***

Forward primer: 5‘-CCTGGATACCGCAGCTAGGA-3‘

Reverse primer: 5‘-GCGGCGCAATACGAATGCCCC-3‘

Product size: 112 base pairs

***GAPDH (glyceraldehyde-3-phosphate dehydrogenase):***

Forward primer: 5‘-CTGCACCACCAACTGCTTAG-3‘

Reverse primer: 5‘-AGGTCCACCACTGACACGTT-3‘

Product size: 282 base pairs

***EF1-α (EEF1A1, eukaryotic translation elongation factor 1 alpha 1):***

Forward primer: 5‘-CTGGAGCCAAGTGCTAACATG-3‘

Reverse primer: 5‘-CCGGGTTTGAGAACACCAGT-3‘

Product size: 221 base pairs
