# Supplementary material for: WNT5A Encodes Two Isoforms with Distinct Functions in Cancers
Source: PLoS One. 2013 Nov 18;8(11):e80526. doi: 10.1371/journal.pone.0080526 (PMC3832467; doi:10.1371/journal.pone.0080526)
Supplement: Table S2 — Sequences of siRNAs. (DOCX) [file pone.0080526.s008.docx]

**Table S2: Sequences of siRNAs**

### Non-silencing control siRNA:

**siLACZ (siRNA targeting bacterial β-galactosidase):**

Sense strand: 5'-gCggCUgCCggAAUUUACC**dTdT**-3'

Antisense strand: 5'-ggUAAAUUCCggCAgCCgC**dTdT**-3'

**siRFP (siRNA targeting soft coral red fluorescent protein):**

Sense strand: 5'-AgACggUggUCAUUACCUA**dGdT**-3'

Antisense strand: 5'-UAggUAAUgACCACCgUCU**dTdT**-3'

**siLUC (siRNA targeting firefly luciferase):**

Sense strand: 5'-CGUACGCGGAAUACUUCGA**dTdG**-3'

Antisense strand: 5'- UCGAAGUAUUCCGCGUACG**dTdG**-3'

### *WNT5A* siRNA:

**siWNT5A (pan isoforms) (siRNA targeting human *WNT5A* mRNA, exon 3):**

Sense strand: 5'-CCCUGUUCAGAUGUCAGAA**dGdT**-3'

Antisense strand: 5'-UUCUGACAUCUGAACAGGG**dTdT**-3'

**siWNT5A-L (long isoform) (siRNA targeting human *WNT5A* mRNA, exon 1):**

Sense strand: 5'-CCCGGUCGCUCCGCUCGGA**dTdT**-3'

Antisense strand: 5'-UCCGAGCGGAGCGACCGGG**dTdT**-3'

**siWNT5A-S (short isoform) (siRNA targeting human *WNT5A* mRNA, exon 1β):**

Sense strand: 5'-UUCUGGCUCCACUUGUUGC**dTdC**-3'

Antisense strand: 5'-GCAACAAGUGGAGCCAGAA**dTdT**-3'
